# Peer victimisation during adolescence and its impact on wellbeing in adulthood: a prospective cohort study

**DOI:** 10.1186/s12889-021-10198-w

**Published:** 2021-01-15

**Authors:** Jessica M. Armitage, R. Adele H. Wang, Oliver S. P. Davis, Lucy Bowes, Claire M. A. Haworth

**Affiliations:** 1 School of Psychological Science, University of Bristol, Bristol, BS8 1TU UK; 2 MRC Integrative Epidemiology Unit, University of Bristol, Bristol, BS8 2BN UK; 3 School of Economics, Finance and Management, University of Bristol, Bristol, BS8 1TU UK; 4 Department of Population Health Sciences, Bristol Medical School, University of Bristol, Bristol, BS8 1UD UK; 5 The Alan Turing Institute, British Library, London, NW1 2DB UK; 6 NIHR Biomedical Research Centre at the University Hospitals Bristol NHS Foundation Trust and the University of Bristol, Bristol, BS8 2BN UK; 7 Department of Experimental Psychology, University of Oxford, Oxford, OX1 3UD UK

**Keywords:** Victimisation, Wellbeing, Resilience, Adolescence, Depression, ALSPAC

## Abstract

**Background:**

Peer victimisation is a common occurrence and has well-established links with a range of psychiatric problems in adulthood. Significantly less is known however, about how victimisation influences positive aspects of mental health such as wellbeing. The purpose of this study was therefore to assess for the first time, whether peer victimisation in adolescence is associated with adult wellbeing. We aimed to understand whether individuals who avoid a diagnosis of depression after victimisation, maintain good wellbeing in later life, and therefore display resilience.

**Methods:**

Longitudinal data was taken from the Avon Longitudinal Study of Parents and Children, a prospective cohort study based in the UK. Peer victimisation was assessed at 13 years using a modified version of the bullying and friendship interview schedule, and wellbeing at age 23 using the Warwick-Edinburgh Mental Well-Being Scale. The presence or absence of depression was diagnosed using the Clinical Interview Schedule–Revised at 18 years. A series of logistic and linear regression analyses were used to explore relationships between peer victimisation, depression, and wellbeing, adjusting for potentially confounding individual and family factors.

**Results:**

Just over 15% of victims of frequent bullying had a diagnosis of depression at age 18. Victimisation also had a significant impact on wellbeing, with a one-point increase in frequent victimisation associated with a 2.71-point (SE = 0.46, *p* < 0.001) decrease in wellbeing scores aged 23. This finding remained after adjustment for the mediating and moderating effects of depression, suggesting that the burden of victimisation extends beyond depression to impact wellbeing. Results therefore show that individuals who remain partially resilient by avoiding a diagnosis of depression after victimisation have significantly poorer wellbeing than their non-victimised counterparts.

**Conclusion:**

Overall, our study demonstrates for the first time that victimisation during adolescence is a significant risk factor for not only the onset of depression, but also poor wellbeing in adulthood. Such findings highlight the importance of investigating both dimensions of mental health to understand the true burden of victimisation and subsequent resilience. In addition to the need for interventions that reduce the likelihood of depression following adolescent victimisation, efforts should also be made to promote good wellbeing.

**Supplementary Information:**

The online version contains supplementary material available at 10.1186/s12889-021-10198-w.

## Background

Mental health and wellbeing make up an integral part of an individual’s ability to lead a fulfilling life, yet mental health problems constitute an astounding proportion of the global burden of disease [1]. A serious risk factor for the development of mental ill-health is peer victimisation [2]. Peer victimisation relates to the experience in which an individual is exposed repeatedly to discomfort at the expense of another peer’s behaviour [3]. There is typically a power imbalance between the perpetrator and victim which is often used to distinguish peer victimisation from general conflict. Peer victimisation is a frequent occurrence in schools worldwide, with prevalence rates up to 35% [4]. Investigating the extent to which this common yet potentially detrimental experience impacts overall mental health functioning could have significant implications for public health.

### Peer victimisation

Peer victimisation typically involves a power imbalance between the perpetrator and victim and can take several forms; overt victimisation is characterised by physical and verbal acts of aggression, while relational victimisation is characterised by experiences of social exclusion [5]. Both types of peer victimisation experienced in adolescence have shown to have severe and lasting repercussions on mental health [6], with victims at an increased risk of anxiety disorders [7] and depression in early adulthood [8]. One of the largest longitudinal studies to date to explore the impact of peer victimisation on mental health reported that around 29% of the cases of depression in the sample could be explained by victimisation, if this were a causal relation [8]. The study used clinical assessments of depression and was therefore able to make generalisations about the population burden of depression that occurs primarily in those of working age [9]. A large number of potentially confounding factors were also adjusted for, including childhood emotional and behavioural problems, depressive symptoms and bullying perpetration in adolescence, as well as family characteristics. Previous research examining the role of peer victimisation in relation to clinical depression had not considered these confounding factors [10]. Adjusting for them is vital in ruling out the independent effects they may have on mental health issues. One problem with interpreting the findings from this study, and much of the existing literature on the longitudinal outcomes of peer victimisation, lies in their sole focus on psychiatric problems [6]. No study to date has considered how peer victimisation may implicate positive aspects of adult mental health, such as wellbeing.

### The importance of wellbeing

Wellbeing is more than the absence of mental illness [11] but refers broadly to feelings of satisfaction and happiness [12]. The concept of wellbeing has been defined and studied in various ways [13], with some separating wellbeing into components known as hedonic and eudaimonic wellbeing [14]. Hedonic wellbeing relates to happiness and pleasure attainment, while eudaimonic wellbeing describes optimal functioning and self-realisation. Within the context of public health, mental wellbeing is often the term adopted to refer to both dimensions of wellbeing [15, 16]. Despite discrepancies in approaches to the study of wellbeing, its role in promoting better physical and mental health is widely acknowledged in both research [17] and policy [18].

Studies exploring the impact of peer victimisation on wellbeing are few, and those that do exist have focused on assessments of hedonic wellbeing in adolescence [19]. While these findings have shown that being victimised by peers is a serious risk factor for lower levels of happiness [19] and a lower quality of life [20] in adolescence, it is not known whether effects generalise to predict wellbeing in adulthood, nor whether there is an interaction with experiencing symptoms of depression. Previous studies focused on predictors of adult wellbeing have revealed long-lasting effects of experiences in childhood and adolescence [21, 22]. It therefore seems likely that peer victimisation in adolescence will continue to influence wellbeing in adulthood. Extending existing findings to understand the extent to which adult wellbeing may be affected by adolescent victimisation could prove crucial to the development of preventive programmes that target victims early to help promote positive development. This is particularly important as the current lack of longitudinal research linking peer victimisation to later wellbeing may be obscuring the true burden of victimisation.

### Resilience

Investigating whether peer victimisation impacts not only the risk of mental health problems, but adult wellbeing will also aid our understanding of how resilience might be attained. Resilience describes the ability to adapt successfully and experience positive functioning following adversity [23]. Despite the emphasis on positive adaptation, however, few have advocated for the importance of understanding how wellbeing may be implicated after an adverse event [24], with most studies incorporating wellbeing concerned with post-traumatic growth [25]. Post-traumatic growth describes the process of benefitting from an adverse event [26], with research often focused on the reappraisals and cognitions of the individual in relation to the experienced event [27]. Resilience on the other hand, is concerned with the processes that allow an individual to move forward and experience positive adaptation following adversity. This adjustment is not necessarily a direct result of the adverse event, nor does it revolve around a significant transformation of the individual [27]. Post-traumatic growth and resilience can thus be viewed as related, but distinct concepts, with evidence to suggest that resilience may moderate the likelihood of post-traumatic growth following trauma [28]. Identifying factors that bolster resilience could thus prove key to cultivating further positive outcomes.

Much of the resilience literature to date has focused on how mental health problems can be avoided [29]. Although mental health problems like depression are related to wellbeing, with correlations between them previously reported at 0.57 [30], they are not completely overlapping dimensions and have different predictors and correlates [31]. For example, individuals may exhibit few signs of a mental disorder but still have a poor quality of life [32]. It is therefore vital that investigations into predictors of mental health consider both wellbeing and mental illness to ensure interventions are equipped to suitably support individuals to foster resilience.

### Current Study

The primary goal of the present study was therefore to extend previous findings focused on depression outcomes in the current sample [8] to explore the impact of adolescent victimisation on adult wellbeing. Our aim was to understand whether individuals who avoid a diagnosis of depression after victimisation, are maintaining good wellbeing later in life, and therefore displaying resilience. It was anticipated that individuals who were subjected to frequent victimisation as an adolescent, but who later avoided depression, would display similar levels of wellbeing to those with no experiences of victimisation or depression. Individuals who were frequently victimised as an adolescent and subsequently depressed in early adulthood were predicted to display lower wellbeing than those victimised but not depressed. This is based on findings of a negative correlation between the two traits, depression, and wellbeing [33]. A second goal of our study was to explore underlying paths linking peer victimisation to later mental health. Understanding possible pathways from an exposure to an outcome is vital to elucidating the direction and strength of effects and how these may vary [34]. We aimed to achieve this through study of the possible mediating and moderating effects of depression on associations between victimisation and wellbeing. In doing so, we hoped to provide insight into the extent to which the impact of victimisation on wellbeing results from an increased risk of depression, which is both predicted by victimisation and negatively associated with wellbeing. To further scrutinise the relationship between victimisation and wellbeing, we also explore the longitudinal impact of victimisation by investigating whether effects on adult mental health remain after accounting for experiences of victimisation in adulthood. Through addressing these aims we hope to provide a more integrated depiction of the true burden of adolescent victimisation and shed further light on what it means to be resilient.

## Methods

### Participants

The sample comprised of participants from the Avon Longitudinal Study of Parents and Children (ALSPAC), a transgenerational prospective study that examines influences on health and development across the life span [35]. Pregnant women resident in a defined region in the South West of England with an expected delivery date between April 1991 and December 1992 were recruited during pregnancy [36, 37]. The initial cohort consisted of 14,062 live births but has since increased to 14,901 children who were alive after 1 year with further recruitment [38]. Follow-up research has largely focused on the offspring, with data collected using a variety of methods including biological samples, clinic assessments, questionnaires, and interviews. Data gathered from 22 years and onwards were collected and managed using REDCap electronic data capture tools hosted at the University of Bristol [39, 40]. Please note that the study website contains details of all the data that is available through a fully searchable data dictionary and variable search tool (http://www.bristol.ac.uk/alspac/researchers/our-data/).

Ethical approval was obtained from the ALSPAC Law and Ethics Committee. Participants included in our study were a subsample of offspring who attended the 13-year research clinic and provided peer victimisation data (*n =* 6529). Of these, 2521 (38.6%) participants provided data for relevant confounding variables and completed the depression assessment at the 18-year research clinic. One thousand four hundred eighty-six of these individuals also completed the wellbeing assessment aged 23. Missing data in our study was most problematic for our confounding variables that related to family characteristics (see Supplementary Table 1, Additional file 1). This is not surprising given the longitudinal nature of our study and the large gap between the assessment of these confounders and our wellbeing outcome. Attrition within the cohort has occurred for various reasons [36], however individuals with complete data within the current sample were no more likely to be a victim of bullying than those lost at follow-up (odds ratio 1.00, 95% confidence interval 0.99 to 1.03, *p* = 0.62). The impact of response attrition, however, was explored in our study using multiply imputed data. Further information regarding sample size can be found in Supplementary Figure 1, Additional file 2.

### Materials

#### Peer victimisation in adolescence

Data were available for peer victimisation at the 13-year clinic, administered using the modified version of the bullying and friendship interview schedule [41]. Adolescents rated the frequency of statements relating to overt and relational victimisation on a 4-point Likert scale (0 = Never, 1 = Seldom, 2 = Frequently, 3 = Very Frequently). All items are based on experiences within the past 6 months. Five items related to overt victimisation and 4 to relational experiences. An example of an item relating to overt experiences is “Someone threatened or blackmailed teenager”, and for relational victimisation, “Peers would not hang around just to upset teenager”. Scores from the overt victimisation items are moderately correlated with the relational victimisation items (r = 0.52) and were therefore examined together. A full list of these items can be found in Supplementary Table 2, Additional file 3. Cronbach’s alpha was calculated across all items and demonstrated good internal consistency (α = 0.73). Overall scores when summed range from 0 to 25 (mean = 1.82, SD = 2.76). A three-level ordinal victimisation variable was constructed to investigate a possible dose-response pattern, as per previous research using this victimisation scale [7, 8]. Adolescents scoring 0 (*n* = 3026) were categorised as ‘Never Victimised’, those scoring between 1 and 3 (*n* = 2361) were ‘Occasionally Victimised’, and individuals scoring 4 or more (*n* = 1145) were ‘Frequently Victimised’. These were coded as 0, 1, and 2 respectively. Using this three-level variable allowed investigations to compare experiences among highly victimised individuals to non-victims.

Mother reports of their child’s victimisation were also recorded when the study child was 12 years of age. Mothers were asked whether their child had often been bullied or picked on by other children in the last 6 months, responding either not true (*n* = 5555, 79.1%), somewhat true (*n* = 1257, 17.9%) or certainly true (*n* = 211, 3%). The inter-rate agreement between the self-reports of victimisation and the mother reports was low (k = 0.04), replicating previous findings [8]. It is important that data on victimisation is gathered from multiple informants to reduce the potential for bias that may arise from certain reporters [42].

#### Peer victimisation in adulthood

In addition to reports of victimisation during adolescence, peer victimisation was also measured when subjects were aged 23. This was assessed using two items that summarised the direct and indirect bullying experiences captured in the 13-year victimisation scale. Participants were asked to rate the frequency that they had repeatedly experienced “having things stolen; being threatened; being blackmailed; called nasty names, had tricks played on them, been hit or shoved”, and were asked about their experiences of indirect victimisation, including “being deliberately left out of get-togethers, parties, trips/groups; being ignored, no longer wanted at the table”. Both statements related to experiences within the last 6 months and responses included ‘Never’, ‘Not Much’ (1–3 times) ‘Quite a lot’ (4 or more times) or ‘A lot’ (at least once a week). Answers to the two statements were moderately correlated (r = 0.40) and had sufficient reliability (internal consistency as measured by Cronbach’s α = 0.54). Responses were combined to assess the impact of overall victimisation in early adulthood.

#### Wellbeing

Wellbeing was assessed for the first time when the ALSPAC cohort were aged 23. Our primary outcome was the Warwick-Edinburgh Mental Well-Being Scale (WEMWBS) [43] because of its widespread use within public health research and its ability to capture overall mental wellbeing. The WEMWBS consists of 14 positively worded items. Individuals are required to choose from a 5-point Likert scale that best describes their experience of that statement over the last 2 weeks. Scores for all items are summed, producing a minimum score of 14 and a maximum score of 70, with a higher overall score reflecting a higher level of mental wellbeing. The internal consistency of this scale in our study was high (α = 0.93), reflecting previous reports [43]. Previous findings have also indicated high test-retest reliability (0.83) [44], verifying the robustness of the scale.

WEMWBS has proven a valid and reliable assessment of overall wellbeing among populations across Europe [44]. However, to explore the specificity of the link between victimisation and wellbeing, follow-up analyses were conducted using measures that capture different components of wellbeing. We include the Satisfaction with Life Scale [45] as a measure of both hedonic and eudaimonic wellbeing, and the Subjective Happiness Scale [46] to tap into hedonic wellbeing only. To explore eudaimonic wellbeing, we include the Meaning in Life Scale [47]. We also explore the link between peer victimisation and the Basic Psychological Needs Scale [48], this captures feelings of autonomy, competence, and relatedness. Information about how these scales correlate with the main outcome measures and each other can be found in Supplementary Table 3, Additional file 4.

#### Depression

At the 18-year clinic (mean age 17 years 10 months), participants completed a self-administered, computerised version of the Clinical Interview Schedule-Revised (CIS-R) [49]. The CIS-R is an interview schedule that establishes the severity of symptoms and diagnoses the presence of a depressive episode according to the International Statistical Classification of Disease (ICD-10) criteria. The CIS-R contains 14 sections, each relating to a certain type of neurotic symptom. Depending on the symptom being assessed, participants were asked about their experience of that symptom in the past 1 week to 1 month. Each symptom is scored from either 0 to 4, or 0 to 5, and contributes towards an overall total score. This score is then used to derive a categorical diagnosis according to the ICD-10. The Cronbach’s alpha for the CIS-R indicated good internal consistency (α = 0.77) and the assessment has proven reliable when administered by a trained interviewer or when self-completed [50]. Further information about the CIS-R is reported elsewhere [44], and its correlation with other variables can be found in the supplementary (Additional file 4).

#### Confounders

The study controlled for several confounding factors (see Supplementary Table 4, Additional file 5 for full details) that have previously been associated with adolescent victimisation [8]. These include depressive symptoms and bullying perpetration, both of which were assessed at the same time as the 13-year victimisation scale, childhood behavioural and emotional problems, child maltreatment (no or present), maternal depression, maternal education, and social class. The present study also controlled for current employment and income within the wellbeing analyses as both have previously been considered as predictors of adult wellbeing [51, 52]. All analyses controlled for sex as there are known differences in the prevalence of depression [53], and evidence to suggest differences in wellbeing between sexes [54].

### Statistical analyses

Logistic regressions were first used in an attempt to replicate previous findings of an association between peer victimisation at age 13 and depression at age 18 [8], adjusted for potentially confounding variables. These models used the three-ordinal variable from the self-reported victimisation scale and were then repeated using mother reports, as per previous research [8]. A series of linear regression models then explored possible relationships between peer victimisation, clinical depression, and mental wellbeing. We first tested for a possible association between peer victimisation at 13 years (using both the self-reports and mother reports), and wellbeing aged 23, without adjustment for confounding factors. Subsequent models then explored the robustness of the relationship through inclusion of the confounding variables, and clinical diagnoses of depression. These adjusted and unadjusted analyses were carried out using separate subsamples of participants to maximise available data and avoid the potential for bias that may arise from using complete cases. We do, however, repeat analyses using complete cases across models and present these in the supplementary (see Supplementary Table 5, Additional file 6).

Including depression as a covariate allowed us to test whether the relationship between peer victimisation and wellbeing is independent of the depressive state of the individual at 18 years. To further explore possible mediating effects of depression, we used the “mediate” function within the mediation R package [55]. This allowed us to calculate Average Causal Mediation Effects (ACME), Average Direct Effects (ADE), as well as combined direct and indirect effects (Total Effects) for the two fitted models. The first model fitted was the mediator model, which refers to the conditional distribution of the mediator given the exposure. The second was the outcome model which is the conditional distribution of the outcome given the exposure and mediator. In addition to investigating mediating effects, we also explore the possible moderating role of depression by subsequently running a regression model predicting wellbeing using an interaction term (victimisation by depression). Finally, analyses examined the longitudinal impact of adolescent victimisation by adjusting for experiences of victimisation in adulthood.

Although missing data was not associated with the likelihood of experiencing victimisation, participants with missing data in ALSPAC are more likely to come from socially advantaged backgrounds [36]. Analyses were therefore repeated following multiple imputation. Multiple imputation using Chained Equations (MICE) [56] was used to simulate multiple values to impute those missing. This was made possible by the wealth of variables available in ALSPAC that predict missingness [7, 8]. Various sociodemographic and mental health variables that have previously been shown to predict attrition were used [36], a full list of which can be found in Supplementary Table 4, Additional file 5. Variable estimates were averaged over 60 imputed datasets based on Rubin’s rules [57] to align with previous procedures on this sample [8]. All analyses were conducted in R Studio version 3.5.1 [58].

## Results

### Descriptive data

At the 13-year research clinic, 6529 participants completed the victimisation assessment. Characteristics of those reporting either no, occasional, or frequent victimisation are presented in Table 1. Individuals who reported frequent victimisation were more likely to report concurrent depressive symptoms in adolescence, and were more likely to have experienced maltreatment and emotional and conduct problems as a child compared to their non-victimised peers. Among individuals that reported some experience of victimisation in adolescence, just under 2% also reported experiences of victimisation in adulthood, while approximately 51% experienced victimisation in adolescence but not adulthood. Just 0.5% of participants experienced victimisation in adulthood only, and around 46.5% of the sample never experienced any victimisation in adolescence or adulthood.

**Table 1:** Sociodemographic and individual characteristics of participants by peer victimisation at 13 years. Values are means (standard deviations) unless stated otherwise

	No Victimisation (***n*** = 3026)	Occasional Victimisation (***n*** = 2360)	Frequent Victimisation (***n*** = 1143)	***p*** value^**a**^
**Individual Characteristics**				
Male (%)	50.5	47.1	47.2	0.03
Childhood emotional problems	1.4 (1.6)	1.5 (1.7)	1.6 (1.7)	< 0.001
Childhood conduct problems	1.4 (1.4)	1.5 (1.4)	1.7 (1.5)	< 0.001
Adolescent depressive symptoms	2.5 (2.6)	4.2 (3.5)	7.2 (5.0)	< 0.001
Adolescent bullying perpetration	0.2 (0.7)	0.8 (1.4)	2.1 (2.4)	< 0.001
**Family Characteristics**				
Lower maternal social class, (%)	21.6	20.0	18.8	< 0.01
Maternal education: O levels or less (%)	58.5	54.9	54.4	0.04
Maternal depression	5.3 (3.7)	5.8 (3.9)	6.1 (3.8)	< 0.001
Maltreatment (%)	2.0	3.0	5.1	< 0.001

Note:

^**a**^ *p* value reflects comparisons between non-victims and victims of frequent victimisation

Of those who completed the victimisation measure at 13 years, 3796 (58.1%) attended and completed the depression assessment at the 18-year research clinic, and 2521 provided information on the relevant confounding variables. 55.5% of these individuals were female and 7.3% had a diagnosis of depression, of which 75% were female. Frequent victimisation was experienced by approximately 17.7% of this sample. Among those who also provided wellbeing data at age 23 (*n* = 1486), 63.5% were female and 16.1% reported frequent victimisation in adolescence. Of these 1486 individuals, 6.5% had a diagnosis of depression. Depression status at 18 years was shown to be a significant predictor of wellbeing at 23 years, with cases of depression associated with a 4.66 (SE = 0.89, *p* < 0.001) decrease in wellbeing scores. After adjustment for peer victimisation and all confounding variables, this association remained, with depression predictive of a 3.30 (SE = 0.89, *p* < 0.001) decrease in wellbeing aged 23. Cases of depression were significantly more likely to be female (86.5%) among those with complete data (*n* = 1486), but average mental wellbeing scores did not significantly differ between males (M = 50.0, SD = 8.52, range = 16–70.) and females (M = 49.39, SD = 8.64, range = 14–70). To explore whether mental health varied among males and females exposed to peer victimisation, we ran regression models predicting depression and wellbeing using an interaction term (victimisation by sex). Findings revealed no moderating impact of sex on the likelihood of experiencing depression following victimisation (*p* = 0.14) but provided some evidence to suggest that females experience lower levels of wellbeing after victimisation than males (*p* = 0.03). Such findings reinforce the importance of appropriate adjustment for sex in our regression models.

### Association between victimisation and depression

Our first set of analyses were a replication of the previous study on the association between peer victimisation and depression [8]. Prior to conducting our logistic regression, we subset participants based on their victimisation status (none, occasional, frequent) and examined the proportion of individuals that were depressed. This allowed comparisons with previous estimates reported in this sample [8]. The presence of depression was shown to be higher among those exposed to more frequent experiences of victimisation. Around 15% of individuals who were frequently victimised as an adolescent were clinically depressed at age 18, compared to 5.6% of those not victimised. Such findings closely align with previous reports in this sample [8].

Logistic regressions examining the association between peer victimisation and depression revealed that the increased risk of depression corresponds to an odds ratio of 2.92 (95% confidence interval 2.17 to 3.93) compared to those who were not victimised (Table 2), with similar findings reported when using mother reports of victimisation (OR = 2.19, 95% confidence interval 1.14 to 3.86). These estimates are highly similar to the previous study [8], with previous reports showing that the increased risk of depression among those self-reporting frequent victimisation corresponds to an odds ratio of 2.96 (95% confidence interval 2.21 to 3.97) compared with those who were not victimised. After adjusting for confounding variables, our analyses using self-reported victimisation were reduced to 1.87 (95% confidence interval 1.18 to 2.95), and the relationship was no longer significant using the maternal reports (OR = 1.80, 95% CI = 0.76 to 3.80), a finding that was also observed previously [8] Results using the imputed dataset showed a similar pattern of results across analyses (Table 2). It should be noted that estimates using both the complete case and imputed datasets vary slightly from previous analyses on this sample [8] due to changes in sample size and variables used for imputation***.***

**Table 2:** Odds ratios for depression at age 18 based on victimisation at age 13 years

		Unadjusted odd ratios (95% CI)		Odd ratios (95% CI)		
Victimisation status	No (%) depressed	All available data (***n*** = 3796)	Complete cases (***n*** = 2521)	Adjusted (***n*** = 2521)^a^	Unadjusted using imputed dataset^b^	Adjusted using imputed dataset^b^
None	1734 (5.6)	1.00	1.00	1.00	1.00	1.00
Occasional	1409 (7.0)	1.26 (0.95–1.68)	1.11 (0.73–1.69)	0.86 (0.58–1.26)	1.14 (0.63–1.55)	1.03 (0.77–1.37)
Frequent	656 (14.9)	2.92 (2.17–3.93)	2.81 (1.95–4.59)	1.87 (1.18–2.95)	2.23 (2.10–3.98)	1.80 (1.22–2.43)

Note:

^a^ Adjustments: children’s individual characteristics (sex, emotional and behavioural problems aged 7, concurrent depressive symptoms and concurrent bullying perpetration aged 13) and family characteristics (social class reported by mothers, mother’s education, maternal depression and child maltreatment experiences between the ages of 5 and 7)

^b^ Missing confounders and additional sociodemographic variables used for imputation (*n* = 4040)

### Association between victimisation and wellbeing

**Fig. 1:**
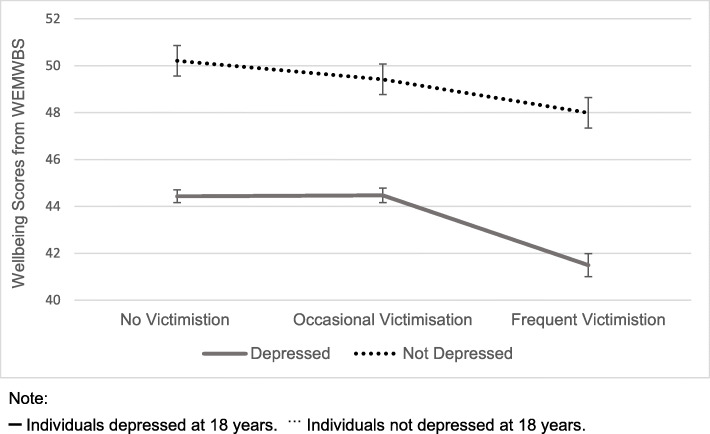
Wellbeing scores based on experiences of peer victimisation aged 13 and depression at 18 years

To test the primary aim of our study, we subsequently explore associations between peer victimisation and adult wellbeing. The wellbeing of individuals who reported frequent victimisation during adolescence was found to vary depending on whether or not individuals received a diagnosis of depression at 18 years, *t*(63.37) = −4.5027, *p* < 0.001. Those who avoided a diagnosis of depression had significantly higher wellbeing at 23 years compared to those who reported depression at 18 years of age. Individuals who experienced frequent victimisation and avoided depression however, had significantly lower wellbeing than individuals with no experiences of either victimisation or depression, *t*(530.89) = − 3.9926, *p* < 0.001. Across all cases, wellbeing was worse for those who were diagnosed with depression compared to those who were not. These findings are represented in Fig. 1.

Linear regression models investigating a possible relationship between peer victimisation and wellbeing revealed that increases in experiences of victimisation are also associated with adult wellbeing. A one-point increase in frequent victimisation reported by the adolescent was associated with a 2.71 (SE = 0.46, *p* < 0.001) decrease in wellbeing scores (Table 3). Similar findings were found when using mother reports, with a one-point increase in victimisation associated with a 2.95 (SE = 1.06, *p* < 0.01) decrease in their child’s wellbeing aged 23. After adjustment for the confounding variables, associations remained significant using the maternal reports of victimisation but not the self-report measures.

**Table 3:** Linear regression results for wellbeing aged 23 years based on experiences of peer victimisation

		Occasional victimisation†				Frequent victimisation††			
	N	Estimate	SE	*P* value	R Squared	Estimate	SE	*P* value	R Squared
**Model 1**									
Unadjusted model	3015	−1.04	0.36	< 0.01	0.01	−2.71	0.46	< 0.001	0.01
**Model 2** ^**a**^									
Adjusted for confounders only	1882	0.08	0.45	0.85	0.08	−0.83	0.67	0.21	0.08
**Model 3** ^**b**^									
Adjusted for depression only	2268	−0.74	0.40	< 0.05	0.04	−2.28	0.53	< 0.001	0.04
**Model 4** ^**a,b**^									
Adjusted for depression and confounders	1486	0.40	0.50	0.42	0.08	−0.53	0.75	0.48	0.08
**Model 5** ^**c**^									
Adjusted for adult victimisation only	2558	−0.90	0.38	< 0.05	0.03	−2.21	0.50	< 0.001	0.03
**Model 6** ^**a,c**^									
Adjusted for adult victimisation and confounders	1631	0.18	0.47	0.71	0.08	−0.44	0.72	0.54	0.08
**Model 7** ^**b,c**^									
Adjusted for adult victimisation and depression	1937	−0.58	0.42	0.17	0.04	−1.94	0.57	< 0.001	0.04
**Model 8** ^**a,b,c**^									
Adjusted for adult victimisation, depression, and confounders	1485	0.55	0.49	0.26	0.10	−0.35	0.74	0.64	0.10

Note:

^**a**^ Adjustments: children’s individual characteristics (sex, emotional and behavioural problems aged 7, depressive symptoms and bullying perpetration aged 13, employment status and income aged 23) and family characteristics (social class reported by mothers, mother’s education, maternal depression and child exposure to physical or sexual abuse aged 7)

^**b**^ Adjustments: depression diagnoses from the CIS-R at 18 years

^**c**^ Adjustments: peer victimisation at 23 years

† Estimates relate to the impact of occasional victimisation on wellbeing aged 23

†† Estimates relate to the impact of frequent victimisation on wellbeing aged 23

To first explore the potential mediating role of depression, analyses were repeated after controlling for depression diagnoses at age 18 (see model 3, Table 3). While slightly attenuated, overall results using both the main and imputed dataset (see Supplementary Table 6, Additional file 7) were highly similar to analyses without depression as a confounder, suggesting that victimisation may exert a significant and direct impact on adult wellbeing. Analyses used to formally test for possible mediation revealed no significant indirect effects of victimisation on wellbeing through depression (ACME = − 0.49, *p* = 0.48), but did identify significant direct and indirect effects of victimisation on wellbeing (Total effects = − 0.79, *p* = 0.05). These are likely driven by the direct effects of victimisation which reached near significance (ADE = − 0.74, *p* = 0.05). Such analyses suggest that the impact of victimisation on wellbeing could be independent of depression.

To further explore the underlying path from peer victimisation to wellbeing, we subsequently examined whether having a diagnosis of depression moderates the impact of victimisation on wellbeing by including an interaction term (victimisation by depression) in the regression model. Findings revealed significant main effects of occasional peer victimisation (β = − 0.80, SE = 0.42, *p* = 0.05), frequent peer victimisation (β = − 2.17, SE = 0.56, *p* < 0.001), and depression (β = − 5.77, SE = 1.09, *p* < 0.001) on wellbeing, but provided no evidence of interactive effects (see Supplementary Table 7, Additional file 8). Such findings suggest that the impact of victimisation on wellbeing is not moderated by depression, meaning that individuals with or without a diagnosis of depression are still likely to experience a reduction in levels of wellbeing.

Finally, to test the robustness of the association between peer victimisation and adult wellbeing, in further analyses we adjusted for experiences of victimisation in adulthood. Although slightly attenuated, results were similar to findings without adult victimisation as a confounder. This was found using both the complete cases (see model 5, Table 3) and imputed datasets (see Supplementary Table 4). Victimisation in adulthood alone was shown to be a significant predictor of levels of wellbeing, associated with a 6.43 (SE = 1.08, *p* < 0.001) reduction in overall wellbeing scores. This was the case even after adjustment for adolescent victimisation and the confounding variables, with adult victimisation predictive of a 5.35 (SE = 1.06, *p* < 0.001) decrease in wellbeing. Thus, the finding that adolescent victimisation remains associated with adult wellbeing even after adjustment for victimisation experiences in adulthood suggests a strong longitudinal impact on adult wellbeing.

### Sensitivity analyses

Beyond these results for our overall measure of mental wellbeing, we conducted follow-up analyses using more specific wellbeing measures. Linear regressions using the Satisfaction with Life Scale [45] revealed the most consistent results to those found using the WEMWBS [43], with frequent victimisation associated with a 2.30 (SE = 0.34, *p* < 0.001) decrease in life satisfaction aged 23. Models that investigated wellbeing using the Subjective Happiness Scale [46] and the Basic Psychological Needs Scale [48] revealed a similar pattern of results, although findings using these scales were attenuated (see Supplementary Table 8, Additional file 9). When investigating the impact on meaning in life aged 23, it was found that this was not predicted by peer victimisation in adolescence.

## Discussion

This study confirmed previous findings of an association between peer victimisation in adolescence and clinical depression in early adulthood [8]. Frequently victimised adolescents were more than twice as likely to be depressed at 18 years compared to non-victimised individuals. We also report for the first time, evidence of an association between adolescent victimisation and adult wellbeing. Individuals subjected to frequent experiences of victimisation during adolescence were at risk for significantly lower mental wellbeing in adulthood. This finding remained even after adjustment for the mediating and moderating effects of the depressive state of individuals at 18 years.

Initial comparisons of wellbeing scores showed that victimised individuals who avoided a diagnosis of depression, and thus who would have been assumed to be resilient and coping well, were actually shown to have poorer wellbeing than their non-victimised counterparts. Such findings demonstrate that the burden of victimisation is worse than what has previously been captured by research focused on depression [8]. It was also noted that at any level of victimisation, wellbeing was lower among individuals that were depressed at 18 years compared to those not depressed. Subsequent analyses exploring this finding further however, revealed no mediating or moderating effects of depression on the relationship between victimisation and wellbeing. This suggests that victimisation may exert its effects on wellbeing through a pathway independent of depression.

Previous studies investigating the impact of victimisation on depression have provided evidence that associations remain after stringent control over confounding variables [8]. However, the extent to which prior mental health problems mediate or moderate these associations has remained largely unknown. The finding that the relationship between peer victimisation and adult wellbeing was not solely explained by indirect mediating effects of depression suggests that victimisation may have a direct impact on wellbeing. This is further supported by the absence of interactive effects of depression which highlight that individuals with or without a diagnosis of depression still experience a reduction in levels of wellbeing. These findings align with previous research on victimisation which have reported that the increased risk of psychopathology among victims is independent of earlier emotional problems [6]. Together these findings reinforce the negative impact of peer victimisation on later mental health and wellbeing and suggest that programs aimed at reducing the prevalence of victimisation in schools could be an effective means to promoting more positive functioning and wellbeing in adult life.

Good wellbeing is both associated with, and precedes important and desirable outcomes, including positive mental and physical health, satisfying work and relationships, and longevity [17]. Understanding early determinants of wellbeing is therefore key in helping to ensure a successful adult life. Our findings suggest that victimisation influences hedonic wellbeing and some aspects of eudaimonic wellbeing, including life satisfaction. Individual autonomy, competence, and relatedness (from the Basic Psychological Needs Scale) were also predicted by adolescent victimisation; however, results suggest that victimisation does not impact meaning in life at age 23. Interventions aimed at improving wellbeing among victims of adolescent bullying should therefore implement strategies that increase overall mental wellbeing as opposed to specific aspects. This wellbeing support should be offered to all victims of bullying, and not just those who receive a diagnosis of depression. This is key as our findings show that individuals who do not meet the criteria for a diagnosis are still in need of psychological support. This is extremely important as there are likely to be fewer resources available to those who do not access formal mental health services. From an epidemiological perspective, the promotion of wellbeing also has the potential to have a greater influence on global mental health than efforts aimed solely at reducing the impact of depression among a smaller minority.

### Strengths and limitations

Our study benefits from several strengths. The high number of potentially confounding variables controlled for is a key strength. Others include the large sample size and the duration of follow-up from the assessment of victimisation in adolescence, to the assessment of wellbeing aged 23. Victimisation was measured using a detailed interview that captured a range of situations. This enabled experiences to be reported that are not readily observable by others, allowing for more accurate estimates. We also supplemented this measure with mother reports of victimisation, and reports of victimisation in adulthood. The finding that associations with wellbeing remained when using mother reports validates our results that relied on self-report measures. By also demonstrating that associations remain after accounting for adult victimisation, our findings extend existing research on the longitudinal consequences of childhood victimisation [59] to establish the long-term impact of adolescent victimisation. Previous longitudinal studies have not accounted for later experiences of victimisation [2], meaning estimates may be slightly inflated. One limitation of our findings, however, is that with the data available we were unable to test for an effect of the accumulation of adversities. Just under 2% of participants were exposed to victimisation in both adolescence and adulthood, meaning analyses would have been underpowered to detect effects of chronic victimisation on wellbeing. An avenue for future longitudinal research could thus be to use larger cohorts to explore the consequences of repeated victimisation across development. Future studies may also wish to explore further the interaction between sex and victimisation identified in our model predicting wellbeing. We note that females had slightly lower wellbeing than males following victimisation. While we adjust for this by including sex as a confounding variable, due to our sample size, we were unable to stratify our sample to explore sex differences following victimisation with sufficient power.

Other potential limitations of our study relate to the generalisability of the ALSPAC cohort. Individuals were born in a defined region in the United Kingdom and as previously noted [8], the loss to follow up from the original ALSPAC sample reflects a slight bias towards individuals from families of a lower social class and educational background (see Table 9, Additional file 10). Participants more likely to complete the peer victimisation, depression and wellbeing assessments in our study were also less likely to have completed earlier measures relating to family socio-economic status and maltreatment (Supplementary Table 1), which may contribute to a potential selection bias. Attrition related to family characteristics and childhood problems, however, have previously shown to have a minimal impact on study estimates in ALSPAC [60]. We also note that analyses using the available data and analyses using the complete case dataset revealed highly consistent findings (Supplementary Table 4). In addition to this, the huge wealth of variables in ALSPAC allowed for multiple imputation with appropriate variables. Analyses using these imputed datasets revealed consistent findings with those carried out using complete cases. The response bias should therefore have a minimal impact on our complete case analyses. Similarly, associations between victimisation and poor psychological adjustment have been well documented in other countries [61, 62], suggesting that while generalisability of the current findings cannot be assumed, it seems likely they will replicate in other cohorts.

Other limitations to note are that models including the confounding factors explained significantly more of the variance in adult wellbeing than peer victimisation alone. This may suggest that the effects of victimisation on wellbeing are dependent upon these factors, raising the question about the role of other unidentified factors that may interact with victimisation to explain adult wellbeing. When interpreting the relationship between peer victimisation, depression, and wellbeing it is also important to consider the possibility of reverse causation. There is concern that the characteristics of victims within the present sample, including increased emotional and behavioural difficulties, may have increased their vulnerability to victimisation [63] and the likelihood that the victimisation is reported. Nevertheless, the association between victimisation in adolescence and wellbeing in adulthood remained after controlling for childhood emotional and behavioural problems, as well as concurrent depressive symptoms using the imputed dataset. This, in addition to the longitudinal design of the current study, helps to reduce the possibility of reverse causality.

Finally, it is important that when interpreting the current findings that the impact of depression beyond 18 years is acknowledged. Some of the impact of victimisation on wellbeing may be explained by the current depressive state of individuals in early adulthood. Later measures of depression were not included in the current study meaning it is not possible to conclude that victimisation has a strong direct impact on wellbeing. It is also important to point out that we focused on resilience using clinical diagnoses of depression. This measure was chosen to ensure results could be compared to previous findings [8] and that resilient functioning could be determined. However, we recognise that this measure does not capture individuals who may report symptoms of depression but do not meet the full diagnostic criteria. Further studies may therefore wish to incorporate more sensitive measures of depression when assessing resilience.

## Conclusions

Overall, our findings demonstrate for the first time that victimisation during adolescence is a significant risk factor for not only the onset of depression, but also poor wellbeing in adulthood. Such effects were not driven solely by an increased risk for depression, highlighting possible direct and negative implications of adolescent victimisation on adult wellbeing. Individuals who remained partially resilient by avoiding a diagnosis of depression following victimisation were shown to have significantly poorer wellbeing than their non-victimised counterparts. It is therefore crucial that wellbeing is assessed and targeted in addition to mental illness after victimisation to attain a more complete understanding of an individual’s intervention needs and to assist them in achieving optimal functioning. This is especially important as up to half of individuals receiving treatment for depression, either in the form of antidepressants or psychotherapy, report a reduction in symptoms [64]. A significant proportion of individuals treated therefore continue to have negative experiences. One way to assist these individuals is to provide wellbeing support. Positive psychological interventions have been shown to not only enhance wellbeing, but also help reduce symptoms of depression [65]. Unlike treatments for depression, wellbeing interventions focus specifically on flourishing and thriving [66], and thus are likely to be more protective against further mental health problems than interventions focused solely on alleviating the mental illness. Such interventions could prove essential to fostering resilience among victims of bullying.

Evidence for the effectiveness of treatments currently delivered to victims of bullying is rare, with most studies focused on how they can reduce the prevalence of bullying [67]. While these preventive measures are both necessary and important, they are yet to eradicate bullying entirely. It is therefore crucial that victims are provided with mental health support and that the effectiveness of different support strategies are evaluated.

Overall, our findings highlight the importance of investigating both dimensions of mental health to understand the true burden of victimisation and subsequent resilience. Further research should now attempt to understand the mechanisms underlying the relationship between peer victimisation and adult wellbeing to provide insight into why some individuals are more likely to experience resilience to victimisation than others. Such investigations should explore biological, genetic, and environmental processes to help elucidate the casual pathways. Research on resilience more generally should also consider whether it is sufficient to avoid a clinical diagnosis, or whether a true marker of resilience is the maintenance of good mental health in addition to the avoidance of mental illness.

## Supplementary Information

**Additional file 1: Supplementary Table 1.** Response patterns across variables.

**Additional file 2: Supplementary Figure 1.** Flowchart of participants in the Avon Longitudinal Study of Parents and Children.

**Additional file 3: Supplementary Table 2.** Frequency of victimisation experiences aged 13. Values are numbers (percentages).

**Additional file 4: Supplementary Table 3.** Pearson correlation estimates among main outcome measures and wellbeing scales.

**Additional file 5: Supplementary Table 4.** Description of confounding factors and variables used for imputation.

**Additional file 6: Supplementary Table 5.** Linear regression results for wellbeing aged 23 years based on experiences of peer victimisation using complete cases (*N*=1485).

**Additional file 7: Supplementary Table 6.** Linear regression results from the imputed dataset for wellbeing aged 23 years based on experiences of peer victimisation.

**Additional file 8: Supplementary Table 7.** Linear regression results from models exploring interactions between victimisation and depression in predicting wellbeing aged 23 years.

**Additional file 9: Supplementary Table 8.** Linear regression results for wellbeing aged 23 years based on experiences of peer victimisation.

**Additional file 10: Supplementary Table 9.** Socio-demographic comparisons of participants with complete and missing data.

## Data Availability

The datasets analysed during the current study are not publicly available as the informed consent obtained from ALSPAC participants does not allow data to be made freely available through any third party maintained public repository. However, data used for this submission can be made available on request to the ALSPAC Executive. The ALSPAC data management plan describes in detail the policy regarding data sharing, which is through a system of managed open access. Full instructions for applying for data access can be found here: http://www.bristol.ac.uk/alspac/researchers/access/. The ALSPAC study website contains details of all the data that are available (http://www.bristol.ac.uk/alspac/researchers/our-data/).

## Abbreviations

SE: Standard error
ALSPAC: Avon Longitudinal Study of Parents and Children
WEMWBS: Warwick-Edinburgh Mental Well-Being Scale
CIS-R: Clinical Interview Schedule-Revised
ICD-10: International Statistical Classification of Disease 10th Revision
MICE: Multiple imputation using Chained Equations
OR: Odds ratio
