# Supplementary material for: Peer victimisation during adolescence and its impact on wellbeing in adulthood: a prospective cohort study
Source: BMC Public Health. 2021 Jan 15;21:148. doi: 10.1186/s12889-021-10198-w (PMC7811215; doi:10.1186/s12889-021-10198-w)
Supplement: Supplementary file 1 — Additional file 1: Supplementary Table 1. Response patterns across variables. [file 12889_2021_10198_MOESM1_ESM.pdf]

Peer victimisation during adolescence and its impact on wellbeing in adulthood: A prospective cohort study.

BMC Public Health

Jessica M. Armitage[a], R. Adele H. Wang, Oliver S. P. Davis, Lucy Bowes, Claire M. A. Haworth.

[a]School of Psychological Science, University of Bristol, Bristol, BS8 1TU, United Kingdom. jessica.armitage@bristol.ac.uk

Supplementary Table 1: Response patterns across variables

	Full sample in ALSPAC (N)	% also with victimisation data [a]	% also with depression data [b]	% also with wellbeing data [c]	% with victimisation, depression, and wellbeing [d]
**Predictor variable**					
Peer victimisation	6529	6529 (100%)	3796 (58.1%)	3015 (46.2%)	2268 (34.8%)
**Outcome variables**					
Depression	4513	3796 (84.1%)	4513 (100%)	2558 (56.7%)	2268 (50.3%)
Wellbeing (WEMWBS)	4041	3015 (74.6%)	2558 (63.3%)	4041 (100%)	2268 (56.1%)
**Confounding variables**					
Individual Characteristics					
Childhood emotional problems	8112	5201 (64.1%)	3548 (43.7%)	3101 (38.2%)	1941 (23.9%)
Childhood conduct problems	8089	5179 (64.0%)	3541 (43.8%)	3103 (38.3%)	1941 (24.0%)
Childhood maltreatment: Physical	8352	5319 (63.7%)	3631 (43.5%)	3173 (40.0%)	1982 (23.7%)
Childhood maltreatment: Sexual	8361	5325 (64.0%)	3634 (43.4%)	3175 (38.0%)	1982 (23.7%)
Childhood maltreatment: Taken into care	8361	5324 (63.7%)	3633 (43.4%)	3175 (38.0%)	1982 (23.7%)
Adolescent depressive symptoms	6627	6467 (97.6%)	3857 (58.2%)	3043 (45.9%)	2250 (34.8%)
Adolescent bullying perpetration	6574	6470 (98.4%)	3824 (58.1%)	3030 (46.1%)	2251 (34.8%)
Income aged 23	3883	2902 (74.7%)	2484 (64.0%)	3811 (98.1%)	2169 (55.6%)
Occupation aged 23	3984	2978 (74.7%)	2535 (63.6%)	3907 (98.1%)	2212 (55.5%)
Family Characteristics					
Maternal social class	9509	4969 (49.4%)	3462 (36.4%)	3043 (32.0%)	1819 (19.1%)

Maternal education: O levels or less	12,311	5980 (48.6%)	4104 (33.3%)	3620 (29.4%)	2115 (17.8%)
Maternal depression	10,269	5610 (54.6%)	3850 (37.5%)	3400 (33.1%)	2031 (19.8%)

[a] Individuals with complete data on the measured variable, who were also assessed for victimisation aged 13.
[b] Individuals with complete data on the measured variable, who were also assessed for depression at the 18 year clinic.
[c] Individuals with complete data on the measured variable, who were also assessed for wellbeing at 23 years
[d] Individuals with complete data on the measured variable, who were also assessed for victimisation aged 13, depression aged 18, and wellbeing at 23 years.
