# Supplementary material for: Peer victimisation during adolescence and its impact on wellbeing in adulthood: a prospective cohort study
Source: BMC Public Health. 2021 Jan 15;21:148. doi: 10.1186/s12889-021-10198-w (PMC7811215; doi:10.1186/s12889-021-10198-w)
Supplement: Supplementary file 3 — Additional file 3: Supplementary Table 2. Frequency of victimisation experiences aged 13. Values are numbers (percentages). [file 12889_2021_10198_MOESM3_ESM.pdf]

Peer victimisation during adolescence and its impact on wellbeing in adulthood: A prospective cohort study.

BMC Public Health

Jessica M. Armitage[a], R. Adele H. Wang, Oliver S. P. Davis, Lucy Bowes, Claire M. A. Haworth.

[a]School of Psychological Science, University of Bristol, Bristol, BS8 1TU, United Kingdom.
jessica.armitage@bristol.ac.uk

Supplementary Table 2: Frequency of victimisation experiences aged 13. Values are numbers (percentages)

	**Frequency of victimisation**			
**Items**	**Never**	**Occasional (1-3 times)**	**Frequent (> 4 times)**	**Very Frequent (>1 per week)**
Someone took teenager's belongings	5173 (77.6)	1101 (16.5)	227 (3.4)	166 (2.5)
Someone threatened or blackmailed teenager	6043 (90.6)	472 (7.1)	99 (1.5)	53 (0.8)
Someone hit or beat up teenager	5906 (88.6)	575 (8.6)	118 (1.8)	67 (1.0)
Someone tricked teenager	6155 (92.2)	438 (6.6)	45 (0.7)	35 (0.5)
Someone called teenager nasty names	4264 (64.1)	1230 (18.5)	589 (8.8)	574 (8.6)
Peers would not hang around just to upset teenager	5976 (89.8)	485 (7.3)	124 (1.9)	68 (1.0)
Peers tried to get teenager to do things he or she did not want to do	6112 (92.0)	416 (6.3)	77 (1.2)	41 (0.5)
Peers told lies about teenager	5533 (83.7)	757 (11.4)	217 (3.3)	104 (1.6)
Peers spoilt games to upset teenager	6332 (95.3)	228 (3.4)	49 (0.7)	40 (0.6)

Note:
Items derived from the modified version of the bullying and friendship interview schedule.
