# Supplementary material for: Peer victimisation during adolescence and its impact on wellbeing in adulthood: a prospective cohort study
Source: BMC Public Health. 2021 Jan 15;21:148. doi: 10.1186/s12889-021-10198-w (PMC7811215; doi:10.1186/s12889-021-10198-w)
Supplement: Supplementary file 4 — Additional file 4: Supplementary Table 3. Pearson correlation estimates among main outcome measures and wellbeing scales. [file 12889_2021_10198_MOESM4_ESM.pdf]

Peer victimisation during adolescence and its impact on wellbeing in adulthood: A prospective cohort study.
BMC Public Health

Jessica M. Armitage[a], R. Adele H. Wang, Oliver S. P. Davis, Lucy Bowes, Claire M. A. Haworth.

[a]School of Psychological Science, University of Bristol, Bristol, BS8 1TU, United Kingdom. jessica.armitage@bristol.ac.uk

Supplementary Table 3: Pearson correlation estimates among main outcome measures and wellbeing scales

	Victimisation	Depression[a]	WEMWBS	Subjective Happiness Scale	Satisfaction with Life Scale	Meaning in Life Scale	Basic Psychological Needs Scale: Autonomy	Basic Psychological Needs Scale: Relatedness	Basic Psychological Needs Scale: Competence
**Victimisation**	1								
**Depression[a]**	0.076***	1							
**WEMWBS**	-0.125***	-0.189***	1						
**Subjective Happiness Scale**	-0.148***	-0.205***	0.702***	1					
**Satisfaction with Life Scale**	-0.120***	-0.164***	0.672***	0.653***	1				
**Meaning in Life Scale**	-0.004	-0.079***	0.248***	0.233***	0.245***	1			
**Basic Psychological Needs Scale: Autonomy**	-0.129***	-0.153***	0.609***	0.570***	0.589***	0.164***	1		
**Basic Psychological Needs Scale: Relatedness**	-0.139***	-0.183***	0.550***	0.583***	0.533***	0.203***	0.664***	1	
**Basic Psychological Needs Scale: Competence**	-0.129***	-0.171***	0.665***	0.628***	0.646***	0.220***	0.688***	0.618***	1

Note:
Correlations drawn from sample of participants with data on victimisation, depression, and all wellbeing measures (n=2096).
[a] Depression diagnoses from the CIS-R at 18 years
* p<0.05, **p<0.01, ***p<0.001
