# Supplementary material for: Peer victimisation during adolescence and its impact on wellbeing in adulthood: a prospective cohort study
Source: BMC Public Health. 2021 Jan 15;21:148. doi: 10.1186/s12889-021-10198-w (PMC7811215; doi:10.1186/s12889-021-10198-w)
Supplement: Supplementary file 5 — Additional file 5: Supplementary Table 4. Description of confounding factors and variables used for imputation. [file 12889_2021_10198_MOESM5_ESM.pdf]

Peer victimisation during adolescence and its impact on wellbeing in adulthood: A prospective cohort study.

BMC Public Health

Jessica M. Armitage[a], R. Adele H. Wang, Oliver S. P. Davis, Lucy Bowes, Claire M. A. Haworth.

[a]School of Psychological Science, University of Bristol, Bristol, BS8 1TU, United Kingdom. jessica.armitage@bristol.ac.uk

Supplementary Table 4: Description of confounding factors and variables used for imputation

Construct	Study child age	Question answered by	Measure name	Number of items	Sample item	Item scoring	Higher score represents	Cronbach's alpha
Confounders								
Bullying Perpetration	13 years	Participant	Bullying and Friendship Interview Schedule[a]	9	"Frequency someone took teenagers personal belongings"	3-point scale ranging from "Seldom" to "Very frequently"	Greater bullying perpetration	0.64
Depressive Symptoms	13 years	Participant	Moods and Feelings Questionnaire[b]	13	"Teenager felt miserable or unhappy"	3-pont scale ranging from "Not at all" to "True"	Greater depressive symptoms	0.84
Emotional problems	7 years	Mother	Strengths and Difficulties Questionnaire[c]	5	"Child often tearful/unhappy in past 6 months"	3-point scale ranging from "Not true" to "Certainly true"	Greater emotional and conduct problems	0.73
Conduct problems	7 years	Mother	Strengths and Difficulties Questionnaire[c]	5	"Child often lied or cheated in past 6 months"			
Child Maltreatment: Physical, sexual or taken into care	7 years	Mother	Child Abuse/Maltreatment	3	"Child was taken into care since his/her 5th birthday"	1=Yes, 0=No	Child has experienced maltreatment	-

Maternal education	32 weeks gestation	Mother	Highest education qualification	1	"What educational qualifications do you have?"	6-point educational scale	Higher educational qualification	-
Maternal depression	21-134 months	Mother	Edinburgh Postnatal Maternal Depressional Scale[d]	10	"Mother has felt sad or miserable in the past week"	4-point scale ranging from "Yes often" to "Never"	Greater depression	0.83
Social class	18 weeks gestation	Mother	Social class based on occupation (mother)	1	-	6-point scale ranging from "Unskilled" to "Professional"	Higher social class	-
Employment	23 years	Participant	Current employment status	4	"Are you currently in full-time paid work (over 30 hours a week)?"	0=No, 1=Yes	Classified as employed if participant is in full-time paid work, part-time paid work, irregular/occasional work, self-employed, or doing modern apprenticeship	-
Income	23 years	Participant	Total take-home pay each month (after tax and national insurance are removed as appropriate)	1	"What is your total take-home pay each month after tax?"	7-point scale ranging from "£0, not in paid work" to "£3000 and above"	Higher income	-
Variables used for imputation								
Mother's age	18 weeks gestation	Mother	Mother's age at first pregnancy	1	"How old were you when you became pregnant for the very first time?"	Responses range from 13 to 46.	Higher age	-
Financial circumstances	32 weeks gestation	Mother	Financial difficulties	5	"Difficulty affording heating"	4-point scale ranging from "Not difficult" to "Very difficult"	Greater financial difficulties	-

Number of pregnancies	33 months	Mother	Number of pregnancies since study child	1	"How many times have you been pregnant since having this study child?"	Responses range from 0 to 9.	Greater number of pregnancies	-
Child IQ	8 years	Participant	Wechsler Intelligence Scale for Children (WISC)[e]		-	Responses range from 45 to 151.	Higher IQ	-
Home ownership	8 weeks gestation	Mother	Home ownership status	1	"Is your home being…"	6-point scale ranging from "Rented from housing association" to "Mortgaged"	Greater home ownership	-
Smoking status	18 weeks gestation	Mother	Daily tar intake pre-pregnancy	4	"Number smoked per day pre-pregnancy"	Responses ranged from 0 to 450.	Greater tar intake	-

[a] Wolke, D., Woods, S., Stanford, K., & Schulz, H. (2001). Bullying and victimization of primary school children in England and Germany: prevalence and school factors. *British Journal of Psychology, 92,* 673-696.
[b] Angold, A., Costello, E. J., Messer, S. C., Pickles, A., Winder, F., & Silver, D. (1995). The development of a short questionnaire for use in epidemiological studies of depression in children and adolescents. *International Journal of Methods in Psychiatric Research*, 5, 237-249.
[c] Goodman, M. R. (2001). Psychometric properties of the Strengths and Difficulties Questionnaire. *Journal of the American Academy of Child and Adolescent Psychiatry, 40,* 1337-1345.
[d] Cox, J. L., Holden, J. M., & Sagovsky, R. (1987). Detection of postnatal depression: Development of the 10-item Edinburgh Postnatal Depression Scale. *British Journal of Psychiatry, 150,* 782-786. DOI:10.1192/bjp.150.6.782
[e] Wechsler, D. (1949). Wechsler Intelligence Scale for Children. San Antonio, TX, US: Psychological Corporation.
