# Supplementary material for: Peer victimisation during adolescence and its impact on wellbeing in adulthood: a prospective cohort study
Source: BMC Public Health. 2021 Jan 15;21:148. doi: 10.1186/s12889-021-10198-w (PMC7811215; doi:10.1186/s12889-021-10198-w)
Supplement: Supplementary file 7 — Additional file 7: Supplementary Table 6. Linear regression results from the imputed dataset for wellbeing aged 23 years based on experiences of peer victimisation. [file 12889_2021_10198_MOESM7_ESM.pdf]

Peer victimisation during adolescence and its impact on wellbeing in adulthood: A prospective cohort study.

BMC Public Health

Jessica M. Armitage[a], R. Adele H. Wang, Oliver S. P. Davis, Lucy Bowes, Claire M. A. Haworth.

[a]School of Psychological Science, University of Bristol, Bristol, BS8 1TU, United Kingdom. jessica.armitage@bristol.ac.uk

Supplementary Table 6: Linear regression results from the imputed dataset for wellbeing aged 23 years based on experiences of peer victimisation.

	**Occasional victimisation†**				**Frequent victimisation††**			
	Estimate	SE	P value	R Squared	Estimate	SE	P value	R Squared
**Model 1**<br>Unadjusted model	-1.10	0.31	<0.001	0.01	-2.76	0.40	<0.001	0.01
**Model 2**<br>Adjusted for confounders only[a]	-0.33	0.32	0.30	0.08	-0.72	0.45	0.05	0.08
**Model 3**<br>Adjusted for depression only[b]	-0.98	0.31	<0.01	0.04	-2.29	0.40	<0.001	0.04
**Model 4**<br>Adjusted for depression and confounders[a,b]	-0.31	0.31	0.32	0.09	-0.53	0.45	0.24	0.09
**Model 5**<br>Adjusted for adult victimisation only[c]	-0.97	0.31	<0.01	0.02	-2.50	0.40	<0.001	0.02
**Model 6**<br>Adjusted for adult victimisation and confounders[a,c]	-0.13	0.24	0.47	0.08	-0.34	0.25	0.31	0.08
**Model 7**<br>Adjusted for adult victimisation and depression[b,c]	-0.89	0.31	<0.01	0.04	-2.11	0.40	<0.001	0.04
**Model 8**<br>Adjusted for adult victimisation, depression and confounders[a,b,c]	-0.24	0.31	0.44	0.09	-0.41	0.45	0.37	0.09

Note:

All results derived from the imputed dataset (N=4040).

[a] Adjustments: children's individual characteristics (sex, emotional and behavioural problems aged 7, depressive symptoms and bullying perpetration aged 13, employment status and income aged 23) and family characteristics (social class reported by mothers, mother's education, maternal depression and child exposure to physical or sexual abuse aged 7).

[b] Adjustments: depression diagnoses from the CIS-R at 18 years.

[c] Adjustments: peer victimisation at 23 years

† Estimates relate to the impact of occasional victimisation on wellbeing aged 23

†† Estimates relate to the impact of frequent victimisation on wellbeing aged 23
