# Supplementary material for: Peer victimisation during adolescence and its impact on wellbeing in adulthood: a prospective cohort study
Source: BMC Public Health. 2021 Jan 15;21:148. doi: 10.1186/s12889-021-10198-w (PMC7811215; doi:10.1186/s12889-021-10198-w)
Supplement: Supplementary file 8 — Additional file 8: Supplementary Table 7. Linear regression results from models exploring interactions between victimisation and depression in predicting wellbeing aged 23 years. [file 12889_2021_10198_MOESM8_ESM.pdf]

Peer victimisation during adolescence and its impact on wellbeing in adulthood: A prospective cohort study.

BMC Public Health

Jessica M. Armitage[a], R. Adele H. Wang, Oliver S. P. Davis, Lucy Bowes, Claire M. A. Haworth.

[a]School of Psychological Science, University of Bristol, Bristol, BS8 1TU, United Kingdom.
jessica.armitage@bristol.ac.uk

Supplementary Table 7: Linear regression results from models exploring interactions between victimisation and depression in predicting wellbeing aged 23 years.

	Wellbeing		
	**Estimate**	**SE**	**P value**
**Main and interactive effects** [a]			
Depression	-5.77	1.10	<0.001
Occasional victimisation	-0.80	0.42	<0.05
Frequent victimisation	-2.17	0.56	<0.001
Occasional victimisation*Depression	0.79	1.59	0.62
Frequent victimisation*Depression	-0.74	1.71	0.67

Note:
[a] This model explored the main and interactive effects of depression and victimisation (N=2268).
