# Supplementary material for: Peer victimisation during adolescence and its impact on wellbeing in adulthood: a prospective cohort study
Source: BMC Public Health. 2021 Jan 15;21:148. doi: 10.1186/s12889-021-10198-w (PMC7811215; doi:10.1186/s12889-021-10198-w)
Supplement: Supplementary file 10 — Additional file 10: Supplementary Table 9. Socio-demographic comparisons of participants with complete and missing data. [file 12889_2021_10198_MOESM10_ESM.pdf]

Peer victimisation during adolescence and its impact on wellbeing in adulthood: A prospective cohort study.

BMC Public Health

Jessica M. Armitage[a], R. Adele H. Wang, Oliver S. P. Davis, Lucy Bowes, Claire M. A. Haworth.

[a]School of Psychological Science, University of Bristol, Bristol, BS8 1TU, United Kingdom. jessica.armitage@bristol.ac.uk

Supplementary Table 9: Socio-demographics comparisons of participants with complete and missing data

	**Complete Case Responders[a] (n=1486)**	**Wellbeing Responders (n=4041)**	**Missing Wellbeing Responders[b] (n=2555)**	**Depression Clinic Responders (n=4514)**	**Missing Depression Responders[c] (n=3028)**	**ALSPAC Sample (n=15443)**	**Missing ALSPAC Sample[d] (n=13957)**
Non-white (%)	3.4	4.1	4.6	4.3	4.7	5.0	5.3
Non-white mother (%)	1.5	2.1	2.5	2.2	2.6	2.6	2.8
Parents own car (%)	97.0	95.2	93.9	95.2	94.0	91.0	89.8
Parents married (%)	87.1	85.8	84.8	83.8	81.9	79.5	78.4
Mother was homeowner (%)	91.1	85.6	81.6	86.2	83.3	77.1	74.9
Mother has University degree (%)	24.5	19.9	16.8	19.1	16.2	12.9	11.3

Note:

[a] Complete case responders have data on wellbeing aged 23, depression aged 18 and all confounding variables.

[b] Individuals with complete data on wellbeing but not depression at 18 years or the confounding variables.

[c] Individuals who completed the CIS-R at the 18-year clinic but do not have data on the confounding variables.

[d] Core singleton ALSPAC sample not in complete case sample.
